# Angiectatic nasal polyps with pleomorphism ‒ a diagnostic pitfall

**DOI:** 10.1016/j.bjorl.2023.101281

**Published:** 2023-06-22

**Authors:** Xiaoli Zhao, Changli Yue, Hongfei Wan, Li Xing, Honggang Liu, Yingshi Piao

**Affiliations:** a Capital Medical University, Beijing Tongren Hospital, Department of Pathology, Dong Cheng District, China; b Beijing Key Laboratory of Head and Neck Molecular Diagnostic Pathology, Beijing, China

**Keywords:** Angiectatic nasal polyps, Sinonasal polyps, Diagnosis, Differential diagnosis

## Abstract

**Objective:**

To generalise the features of PANP in case of potential clinical and pathological pitfall of diagnosis.

**Methods:**

Thirteen patients diagnosed as PANP were retrospectively analyzed in the Pathology Department of Capital Medical University from August 2014 to December 2019. Immunohistochemical staining with CD34, CK, Vim, Calponin, Ki67, Bcl-2, and STAT-6 was performed with envision-two steps method.

**Results:**

PANP is a benign tumor presenting with gross variegated tan to gray soft fleshy tissue with foci of obvious hemorrhage and necrosis. The imaging shows internal heterogeneous hyperintensity with a peripheral hypointense rim while postcontrast images display a strong nodular and patchy enhancement. Vimentin (Vim) stain was consistently positive, while negative for CD34, STAT-6 and Bcl-2 (focal positive in two cases). Calponin and CK stain was positive in nine cases, respectively.

**Conclusion:**

PANP is a clinically rare tumor which may simulate malignancy lesion. Recognizing of characteristic features in these thirteen patients would be beneficial to avoid misdiagnosis and unnecessary aggressive treatment.

**Level of evidence:**

This work was Level 2 of evidence according to the Guide for Authors.

## Highlights

• A very rare type of benign sinonasal polyps.
• Potential misdiagnosis as malignancy.
• Immunohistochemical stains are very contributive to diagnosis.

## Introduction

Sinonasal polyps are morphologically classified into five types – edematous, fibrous, glandular, cystic and angiectatic.^1^ Angiectatic Nasal Polyp (ANP), also known as angiomatous polyps, is a very rare type of sinonasal polyps. Angiectatic Nasal Polyp with Pleomorphism (PANP) are benign lesions of nose and paranasal sinuses,^2^ characterized by hemorrhage and necrosis, occasionally accompanied by bizarre large pleomorphic spindle cells hyperplasia. Clinically, it grows rapidly and exhibits an aggressive clinical behavior, simulating malignancy.^3^, ^4^, ^5^, ^6^ In spite of its characteristic findings on imaging, however, it is radiologically challenging to pick it up. Therefore, histopathology features are essential for making definitive diagnosis.

In this report, we identified and summarized the features of thirteen patients with PANP from clinical manifestations, imaging, and histopathology. Awareness of these features would be helpful to avoid misdiagnosis and unnecessary treatment.

## Methods

### Ethical justification

The study was approved by the Ethical Committee of the Department of Pathology in Beijing Tongren Hospital affiliated to Capital Medical University. We retrospectively analyzed thirteen patients diagnosed as PANP in the Department of Pathology in Beijing Tongren Hospital affiliated to Capital Medical University from August 2014 to December 2019. Clinical data are summarized in Table 1.

**Table 1:** Clinical Data of 13 PANPs.

Patient number	Gender	Age, years	Location	Operation time (yr.mo)	Clinical symptoms	Imaging performance	Prognosis
1	M	11	Posterior nostril of left maxillary sinus	2014.8	Intermittent nasal obstruction with bleeding	MRI showed soft tissue shadow in posterior nostril of maxillary sinus.	Postoperative recovery
2	F	12	Posterior nostril of left maxillary sinus	2014.9	The nasal obstruction aggravated gradually; a runny nose accompanied the olfactory decline, sometimes sneezing.	MRI showed soft tissue shadow in left ethmoid, maxillary sinus and nasal cavity masses.	Postoperative recovery
3	M	35	Left frontal, ethmoid, maxillary sinus	2015.12	The nasal obstruction and a runny nose in the left side had appeared for more than one year.	MRI showed soft tissue shadow in the left frontal sinus, ethmoid sinus and maxillary sinus. The bone in the left inferior wall of maxillary sinus was discontinuous.	Postoperative recovery
4	M	17	Left maxillary sinus	2017.9	Paroxysmal left upper toothache, left progressive nasal obstruction, headache, sneeze, and runny nose.	MRI showed soft tissue shadow in left maxillary sinus and nasal cavity. The bone was discontinuous in medial wall, posterior wall, and parietal wall of maxillary sinus.	Postoperative recovery
5	M	7	Left nasal cavity	2017.1	Bilateral nasal obstruction with runny nose for more than 20-days.	MRI showed soft tissue shadow in left upper and middle nasal meatus.	Recurred after five months and recovered postoperative
6	M	11	Right nasal cavity	2017.9	Runny nose for two years, stuffy nose for more than one month, with headache, severe on the right.	CT showed soft tissue shadow in the right nasal cavity and paranasal sinus, which involved local bone of the right maxillary sinus.	Postoperative recovery
7	F	13	Left nasal cavity and paranasal sinus	2018.1	Left nasal obstruction for one year and left nasal swelling for more than half a year.	MRI showed soft tissue shadow in the left nasal cavity and nasopharynx, widely involving the surrounding structures.	Postoperative recovery
8	M	9	Left nasal cavity	2018.3	Left intermittent nasal obstruction for five months.	MRI showed the soft tissue shadow in the left nasal tract which protruded into the nasopharynx.	Recurred after 1-year and recovered postoperative
9	M	13	Left nasal cavity and maxillary sinus	2019.7	Left persistent nasal obstruction for 3-years and had aggravated for 3-months.	MRI showed soft tissue shadow in left maxillary sinus and nasal cavity.	Postoperative recovery
10	M	15	Left nasal cavity	2019.9	Nasal bleeding marked on the left for more than 2-months.	MRI showed soft tissue shadow in left maxillary sinus and nasal cavity.	Postoperative recovery
11	F	54	Left maxillary sinus and nasal cavity	2019.9	Nasopharyngeal bleeding for half a year.	MRI showed soft tissue shadow in left maxillary sinus and nasal cavity.	Postoperative recovery
12	M	15	Right maxillary sinus	2019.11	Right nose nasal obstruction with purulent discharge had appeared for nine months, and the right eye was swollen with pain for three weeks.	MRI showed soft tissue shadow in right maxillary sinus.	Postoperative recovery
13	M	58	Left maxillary sinus, middle nasal meatus, and posterior nostril	2019.12	Left nasal obstruction with runny nose had appeared for more than 1-year.	MRI showed soft tissue shadow in left maxillary sinus, middle nasal meatus, and posterior nostril.	Postoperative recovery

M, Male; F, Female; yr, years; mo, months; MRI, magnetic resonance imaging; CT, computed tomography.

### Methods

Histology tissue samples were fixed with 10% neutral formaldehyde, dehydrated with ethanol and embedded in paraffin. 4 μm thick sections were prepared and stained with Hematoxylin-Eosin Staining (HE) and Immunohistochemistry (IHC) staining method. The sections were observed under B ×50 microscope of Olympus.

### Hematoxylin-eosin (HE) staining

4 µm sections were obtained from each paraffin block using a microtome (Thermo Scientific, MICROM HM 340E, America) and stained with HE. Samples were de-waxed in two changes of xylene (5 min each), rehydrated to water with graded alcohols and stained with hematoxylin for 5 min. After rinsing the sections in water, the hematoxylins were differentiated in 1% hydrochloric acid in 70% ethanol. Sections were stained with eosin for 3 min, re-immersed in alcohol and xylene, dehydrated in ethanol, cleared in xylene and cover slipped in a resinous mountant.

### Immunohistochemistry

Immunohistochemical staining was performed with envision-two steps method. Markers are mouse anti-human antibodys, including ZM-0046 CD34 (OriGene, 1:100, clone 10C9), ZM-0069 CK (OriGene, 1:200, clone AE1/AE3), ZM-0260 Vim (OriGene, 1:200, clone UMAB159), ZA-0524 Calponin (EPI, 1:150, clone EP63), ZM-0166 Ki67 (OriGene, 1:200, clone UMAB107), ZM-0010 Bcl-2 (Leic, 1:100, clone Bcl-2/100/D5) and ZA-0647 STAT-6 (EPI, 1:150, clone EP325).

Tissue sections were deparaffinized and rehydrated. Antigen retrieval was achieved by pressure cooking in 0.1 M citrate buffer, Ph 6, for 10 min followed by cooling at room temperature before incubation with the antibodies. Sections were pre-incubated with 3% hydrogen peroxide at room temperature for 10 min so as to block nonspecific antibody binding. Subsequently, the sections were incubated overnight at 4 °C with specific primary antibodies followed by horseradish peroxidase-linked sheep anti-mouse/rabbit secondary antibody (Origene, America) for 60 min. Controls were carried out by omitting the first antibody. The reactions were visualized by 3, 3-Diaminobenzidine (DAB). The slides were then counterstained with hematoxylin. Slides were mounted using a synthetic resin.

## Results

### Clinical presentation

**Figure 1:**
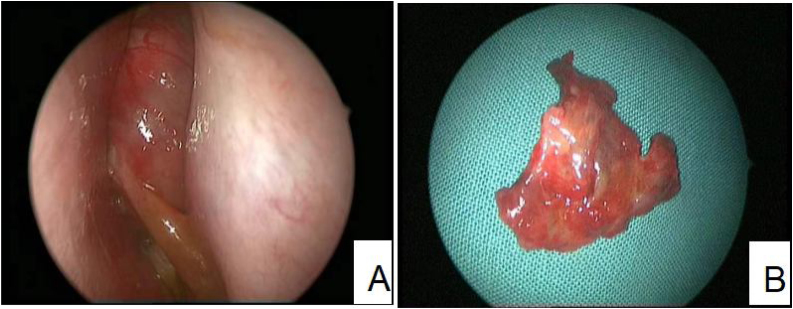
(Case 4) (A) Under nasal endoscopy, a large polypoid mass was seen in the nasal cavity. The mass was soft and smooth, with surface protrusions and vascular dilation. (B) The color of the tumor shown inhomogenous appearance (light red mixed with black and yellow).

**Figure 2:**
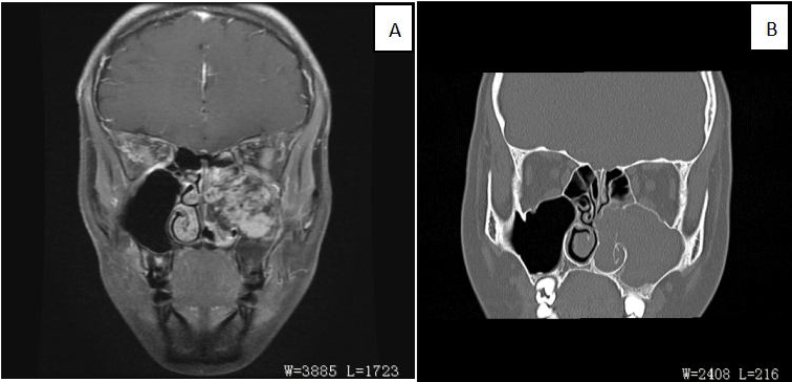
(A) Coronal fat-suppressed contrast-enhanced T1WI showed that the soft tissue mass in the left maxillary sinus displayed uneven and obvious enhancement (red arrow). Bone was discontinuous in medial wall, posterior wall and parietal wall of maxillary sinus. (B) The coronal CT bone window reconstruction image showed an expansion of the left maxillarysinus, increased density in the sinus cavity, and the lesion through the sinus opening into the left nasal cavity. The bone of the left middle and lower turbinates was resorpted, and the boundary with the lesion was unclear.

The average age of thirteen patients with PANP was 20.8 (range 7–58), median age was 13. The gender ratio was 10:3 (male to female) (Details in Table 1). Patients presented nasal obstruction, snoring, headaches, toothache, orbital pain and/or facial pain. Office endoscopic examination revealed a large polypoid mass in the nasal cavity and paranasal sinuses. The mass had an unusual inhomogenous appearance (Fig. 1). Computed Tomography (CT) demonstrated a soft tissue-density mass, with bone discontinuity usually. Magnetic Resonance Imaging (MRI) showed the mass with hypointensity on T1-weighted images and hyperintensity intensity on T2-weighted images.The mass showed markedly heterogeneous enhancement after contrast material administration (Fig. 2).

### Histologic features

**Figure 3:**
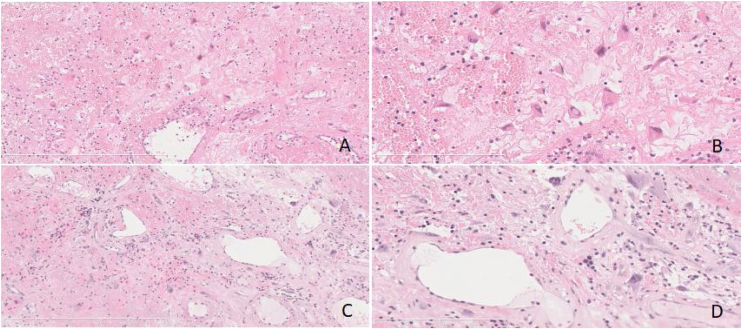
(A‒D) Some stromal cells were enlarged and pleomorphic, the nucleus was abnormal and deeply stained. Obvious dilated vascular components, accompanied by hemorrhage and infarction were also seen in the interstitum. The deposition of pink staining and amorphous protein like substance were present. (Fig. A and C, original magnification ×100, Fig. B and D, original magnification ×200, Hematoxylin and Eosin staining).

Thirteen cases of PANP were composed of variegated tan to gray soft fleshy tissue, while accompanied by obvious hemorrhage and necrosis. Their stroma was edematous and loose mucoid composed of enlarged and pleomorphic cells with atypical hyperchromatic nucleus. Obvious dilated vascular components, sometimes accompanied by hemorrhage, thrombosis and infarction were also seen in the interstitum. The deposition of pink staining and amorphous protein like substance were present occasionally (Fig. 3).

### Immunohistochemistry

As summarized in Table 2, immunohistochemistry stain is another valuable method to diagnosis. Vimentin (Vim) stain was consistently positive, while negative for CD34 and STAT-6. Calponin stain was positive in nine cases. CK stain was positive in nine cases. Bcl-2 stain was focal positive in two cases. All cases showed low Ki67 positive indexes (Table 2).

**Table 2:** Immunohistochemical Expression of Thirteen PANP cases.

Patient number	CK	Vim	Calponin	Bcl-2	STAT-6	CD34	Ki67
1	–	+	–	–	–	–	<1%
2	+	+	–	–	–	–	<1%
3	–	+	+	–	–	–	<1%
4	+	+	+	–	–	–	1‒5%
5	–	+	–	–	–	–	<1%
6	+	+	+	–	–	–	1‒5%
7	–	+	+	Focal+	–	–	1‒5%
8	+	+	+	Focal+	–	–	<1%
9	+	+	+	–	–	–	<1%
10	+	+	+	–	–	–	<1%
11	+	+	–	–	–	–	5‒10%
12	+	+	+	–	–	–	<1%
13	+	+	+	+	–	–	<1%

## Discussion

Angiectatic Nasal Polyp (ANP), a rare inflammatory sinonasal polyp, often develops secondary to changes in choanal polyp. It is vulnerable to vascular compromise in specific sites, such as ostium, posterior end of the inferior turbinate, choana and nasopharynx.^2^, ^7^, ^8^ Nasal obstruction is the most common symptom, followed by decrease or loss of smell perception, epistaxis, proptosis and visual disturbances.^2^, ^7^, ^8^, ^9^

PANP is an exceedingly rare subtype of ANP. The course of the disease is long, the progress is slow, and the bone is swelling rather than erosive destruction. It is often unilateral, manifested as runny blood or epistaxis and accompanied by swelling destruction of the bone wall of the paranasal sinuses. Histologically, it is characterized by extensive vascular proliferation and dilation with Congo red negative pseudoamyloid material deposition.^7^ Shobha et al. found racemose aggregates of irregularly shaped blood vessels resembling dilated capillaries without elastic or muscular layers, accompanied by patchy necrosis and atypical stromal spindle cells.^3^, ^10^, ^11^ Electron microscopy and immunohistochemistry (CD34, factor VIII) results show endothelial cells lining the spaces and myofibroblasts in interstitium.^2^ On the MRI scan, T2-weighted images show internal heterogeneous hyperintensity with a peripheral hypointense rim while postcontrast images display a strong nodular and patchy enhancement.^12^, ^13^ Vessel-like marked and progressive enhancement are important features on 2-phase helical CT scan. It is often associated with a soft tissue mass shadow of uneven density, with stripped and nodular high-density shadow located around and inside the lesion. The adjacent bone, especially the inner wall of maxillary sinus, shows discontinuous compression and absorption changes.

Given the manifestations of imaging and pathology, the diagnosis of PANP is still difficult because of potential confusion with Pleomorphic Hyalinizing Angiectatic Tumor (PHAT). PHAT is one of the low-grade intermediate ‒ locally aggressive soft tissue mesenchymal tumors with undetermined tissue differentiation.^14^ It mainly occurs in adults and is classified as undetermined differentiation tumors in soft tissue and bone tumors by WHO in 2020.^15^ Smith et al. first described its morphology, which is characterized by the expanded hyaline degeneration of the cluster thin-walled vessels, and pleomorphic spindle shaped and oval shaped pleomorphic cells in interstitium.^16^ It is extremely rare and easily misdiagnosed since the morphology is similar to that of PANP. A large number of dilated thin-walled blood vessels are distributed in clusters, with different lumen sizes. Pink staining and amorphous protein like substance can be seen under the intima of the vessels. Hyaline degeneration can be seen on the wall of the vessels, extending from the vessels to the interstitium around the vessels. Organized and recanalized thrombus can be seen in some vessels. Pleomorphic tumor cells are interspersed between blood vessels in sheet or bundle shape. The tumor cells are spindle, round or oval, with pleomorphic nuclei. Giant cells of pleomorphic tumor can be seen, but mitosis is rare. It is a characteristic histological change, with pseudoinclusions in the nucleus. Erbolat KQ et al. thought that vimentin and CD34^17^, ^18^ were positive in PHAT tumor cells, were positive in some of them, and low Ki67 PI suggested that tumor cells had low proliferative activity (<5%).^19^ Imaging findings showed that surrounding bone were damaged, sometimes discontinuously, in both. However, histological examination showed that PHAT appeared to contain more hyalinized and degenerated thin-walled dilated blood vessels and marked stomal atypia, which was helpful for distinguishing.

Complete removal of PANP lesions by operation is the only effective treatment method, and the prognosis is good. On the contrary, PHAT is an intermediate ‒ locally aggressive soft tissue tumor with high recurrence rate. Therefore, extend local resection and long-term follow-up are recommended. In summary, understanding the clinical manifestations, imaging and pathological features are essential for making proper diagnosis and treatments between the two diseases in the nasal cavity and sinuses.

## Conclusion

PANP is a clinically rare tumor which may simulate malignancy lesion. Recognizing of characteristic features in these thirteen patients would be beneficial to avoid misdiagnosis and unnecessary aggressive treatment.

## Funding

There is no funding for this work.

## Conflicts of interest

The author declares no conflicts of interest.
